# 754. A Two-step Testing Algorithm for Hospital-onset *Clostridioides difficile* Infection (CDI) Reduces Prescribing of *C. difficile* (CD) Therapy but Its Ability to Guide Treatment Decisions Remains Unclear

**DOI:** 10.1093/ofid/ofab466.951

**Published:** 2021-12-04

**Authors:** Mackenzie Dolan, Heather Cox, Cirle A Warren, Costi Sifri, Melinda Poulter, Lindsay E Donohue, Amy J Mathers

**Affiliations:** 1 University of Virginia Health, Charlottesville, Virginia; 2 University of Virginia, Charlottesville, Virginia; 3 Office of Hospital Epidemiology/Infection Prevention & Control, UVA Health, Charlottesville, VA, Charlottesville, Virginia; 4 UVA Health, Charlottesville, Virginia; 5 University of Virginia Health System, Charlottesville, VA

## Abstract

**Background:**

Determining true CDI versus CD colonization through CD testing is a continuing challenge. A previously introduced decision support tool at UVA Health significantly reduced inappropriate testing without adverse outcomes. More recently, our methodology changed from nucleic acid amplification test (NAAT) alone to an initial NAAT followed by ELISA for toxin to improve specificity. The purpose of this analysis was to assess provider interpretation of test results, using targeted CD therapy as a surrogate.

**Methods:**

This single-center, retrospective study evaluated all patients with a positive NAAT (Cepheid Xpert® *C. difficile*) on day 4 or later of hospitalization following 2-step algorithm implementation from Feb 2020 through Feb 2021. Toxin negative (TOX-) test results (C. DIFF QUIK CHEK COMPLETE®) were accompanied by a comment that discordance may represent colonization or CDI and to consider ID consult. The proportion of toxin positive (TOX+) versus TOX- patients receiving ≥ 1 dose of CD therapy served as the primary outcome with partial courses considered < 10 days. Clinical outcomes were also compared.

**Results:**

Ninety patients with NAAT+ results were included, of whom 58 (64%) were TOX-. Thirty-two (100%) TOX+ (median days of therapy [IQR] = 14 [11-17]) versus 51 (88%) TOX- patients (median days of therapy [IQR] = 11 [7-14]) received CD therapy (p=0.04). Treatment decisions were guided by ID physicians for 32 (63%) TOX- patients; ID recommendations to discontinue CD therapy were followed in 2 out of 9 (22%) cases. TOX- patients received partial therapy due to patient death (n=5), presumptive colonization (n=3), and provider error (n=1). Of TOX- patients receiving partial or no treatment, there were no CDI-related adverse outcomes during the admission. CDI-related colectomy occurred in 2 (6%) and 1 (2%) TOX+ and TOX- patients, respectively. Five in-hospital deaths with CDI as a contributing factor occurred in the TOX+ group.

**Conclusion:**

Adoption of a 2-step NAAT plus toxin testing algorithm for hospital-onset CDI reduced the frequency with which TOX- patients received CD therapy but the vast majority were still treated. Most providers considered a positive NAAT indicative of CDI, regardless of TOX status.

**Disclosures:**

**All Authors**: No reported disclosures

